# Three new species of the genus *Cymodusa* Holmgren (Hymenoptera, Ichneumonidae, Campopleginae) from Korea

**DOI:** 10.3897/zookeys.311.5529

**Published:** 2013-06-20

**Authors:** Jin-Kyung Choi, Janko Kolarov, Jong-Wook Lee

**Affiliations:** 1 Department of Life Sciences, Yeungnam University, Gyeongsan, 712-749, Korea; 2 Faculty of Pedagogy, University of Plovdiv, 24 Tsar Assen Str., 4000 Plovdiv, Bulgaria

**Keywords:** *Cymodusa aenigma* Dbar, *Cymodusa koreana* sp. n., *Cymodusa yeungnamensis* sp. n., *Cymodusa geolimi* sp. n., taxonomy

## Abstract

Korean species of the genus *Cymodusa* Holmgren (Hymenoptera: Ichneumonidae: Campopleginae) are reviewed. Four species of *Cymodusa (Cymodusa)* are reported from Korea, including one newly recorded species, *Cymodusa aenigma* Dbar (1985), and three new species, *Cymodusa koreana* **sp. n.**, *Cymodusa yeungnamensis* **sp. n.** and *Cymodusa geolimi* **sp. n.** This genus is reported for the first time from Korea. Descriptions with photographs of new species, line drawings of propodeum and metasomal tergites of the Palaearctic species of the “*australis*” group and a key to the Korean *Cymodusa* species are provided.

## Introduction

The genus *Cymodusa* Holmgren (1859) is a moderately large genus in the subfamily Campopleginae Förster with contains about 40 described species from the Eastern Palaearctic, Nearctic, Neotropical, Oriental and Western Palaearctic regions (Yu et al. 2012). Since Holmgren (1859) described *Cymodusa leucocera*, 38 species have been recorded. Recently, Kolarov and Coruh (2008) and Kolarov and Yurtcan (2008) reported two new species from Turkey. In the Eastern Palaearctic region there are 15 species (Yu et al. 2012). This genus usually occurs among grasses and they are parasitoids of Lepidoptera (Kolarov and Yurtcan 2008). One of the collecting sites was clothed in trees, herbaceous plants and shrubs. The Palaearctic species have been reviewed and keyed by Dbar (1984, 1985).

Dbar (1985) recognizes four species groups, namely the “*jaceki*” group, “*leucocera*” group, “*australis*” group and “*convergator*” group. The “*australis*” group includes eight species, *Cymodusa australis* Smits van Burgst (1913), *Cymodusa orientalis* Uchida (1956), *Cymodusa longiterebra* Dbar (1985), *Cymodusa tibialis* Dbar (1985), *Cymodusa rufiventris* Dbar (1985), *Cymodusa aenigma* Dbar (1985), *Cymodusa parva* Dbar (1985) and *Cymodusa oculator* Dbar (1985), in the Palaearctic region as well as three species and one subspecies, *Cymodusa dravida* Gupta & Gupta (1974), *Cymodusa josephi* Gupta & Gupta (1974), *Cymodusa josephi malaise* Gupta & Gupta (1974) and *Cymodusa shiva* Gupta & Gupta (1974), in the Oriental region.

The “*australis*” group can easily be distinguished from the other species groups by the following characteristics: temple very narrowed; areolet with the 2^nd^ recurrent vein before the middle; 6^th^ and 7^th^ tergites deeply emarginate in dorsal view.

In this study, we report the “*australis*” group of *Cymodusa* for the first time from Korea, including one newly recorded species and three new species.

## Materials and methods

Materials used in this work were collected by sweeping and Malaise trapping, and were deposited in the animal systematic laboratory of the Yeungnam University (YNU, Gyeongsan, Korea). Some specimens examined in this study were loaned by the Naturhistoriska Riksmuseet, Sektionen for Entomologi (Swedish Museum of Natural History Department of Entomology) (NR, Stockholm, Sweden). Specimens were examined using a stereo microscope (Zeiss Stemi SV 11 Apo; Carl Zeiss, Göttingen, Germany) and key characters shown in the photographs were produced using a Delta imaging system (i-Delta 2.6; iMTechnology, Daejeon, Korea). The morphological terminology is mostly that of Gupta and Maheshwary (1977). Abbreviations are as follows. TD, type depository; TS, type species; ZI, Zoological Institute, Academy of Sciences, St. Petersburg 199034, Russia; GW, Gangwon-do; GB, Gyeongsangbuk-do; GN, Gyeongsangnam-do.

## Results

### Key to the Korean *Cymodusa*

**Table:** 

1	Costula of propodeum complete (Fig. 5A); spiracle of propodeum very small circle, its diameter shorter than carina linking spiracle to pleural carina (Fig. 1I)	*Cymodusa aenigma*
–	Costula of propodeum incomplete or absent (Fig. 5I); spiracle of propodeum large, its diameter longer than carina linking spiracle to pleural carina (Fig. 2H)	2
2	Mandible blackish brown (Figs 4C and D); areolet of fore wing with stalk (Fig. 4M)	*Cymodusa geolimi* sp. n.
–	Mandible yellowish brown (Figs 3C); areolet of fore wing without stalk (Fig. 3L)	3
3	Spiracle of propodeum large (Fig. 3I); first to third flagellomeres to yellowish brown (Fig. 3M); notaulus absent (Fig. 3F)	*Cymodusa yeungnamensis* sp. n.
–	Spiracle of propodeum small (Fig. 2H); all antennal flagellomeres blackish brown; notaulus present as weak trace (Fig. 2F)	*Cymodusa koreana* sp. n.

### Family Ichneumonidae Latreille, 1802 Subfamily Campopleginae Förster, 1869

#### Genus Cymodusa (Cymodusa)

Holmgren, 1859

http://species-id.net/wiki/Cymodusa

Cymodusa Holmgren, 1859: 327. TS: *Cymodusa leucocera* Holmgren
Thersilia Schmiedeknecht, 1907: 598. TS: (*Thersitia egregia* Schmiedeknecht) = *Cymodusa leucocera* Holmgren.

##### Diagnosis.

Eyes densely setose. Inner margins of eyes strongly convergent ventrally. Clypeus small; apical margin rounded; weakly convex, not separated from face. Mandible short, upper tooth as long as or longer than lower tooth. Hind basitarsus without a median ventral row of close setae. Fore wing with areolet; nervulus opposite to basal vein or distad of it. Thyridium longitudinally elliptic. Ovipositor straight, with dorsal subapical notch.

#### Cymodusa (Cymodusa) aenigma

Dbar, 1985

http://species-id.net/wiki/Cymodusa_aenigma

Figs 1 , 5A , 6A

Cymodusa (Cymodusa) aenigma Dbar, 1985: 589. Holotype: female; TD: ZI.

##### Material examined.

[Korea]: 2 females, Mureung valley, Samhwa-dong, Donghae-si GW, Korea, 15 July–1 August 2005, MT (Malaise trap), J.W. Lee; 1 female, ditto, 9–17 August 2005, MT, J.W. Lee; 1 female, ditto, 31 August-10 September 2005, MT, J.W. Lee.

##### Description.

Female. Body length 6.1 mm.

Fore wing length 3.3–3.8 mm.

Antenna with 34–35 flagellomeres.

*Color*. Head black. Scape and pedicel blackish brown. Mandible yellow, apically brown; labial and maxillary palps pale yellow. Antenna blackish brown. Mesosoma black. Tegula brown. Fore leg yellow; mid leg yellowish brown; hind coxa black, trochanter dark brown, femur and tibia brown, tarsus blackish brown. Metasoma black. Thyridium reddish brown. 5^th^ to 7^th^ metasomal segments reddish brown. Ovipositor yellowish brown.

Morphology.

**Figure 1.**
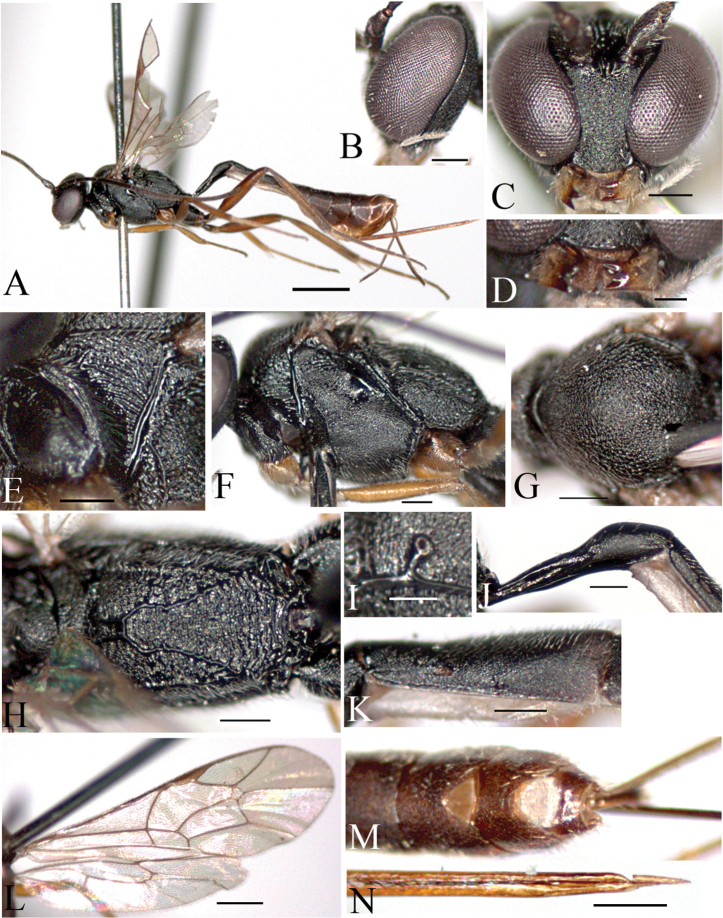
*Cymodusa (Cymodusa) aenigma* Dbar, 1985 (female). **A** habitus in lateral view **B** head in lateral view **C** head in frontal view **D** mandible **E** pronotum **F** mesopleuron in lateral view **G** mesoscutum in dorsal view **H** propodeum **I** spiracle of propodeum **J** petiole in lateral view **K** thyridium **L** wings **M** 6^th^ and 7^th^ tergites in dorsal view **N** ovipositor. (Scale bar 1 mm for **A**; 0.5 mm for **L**; 0.2 mm for **B, C, E–H, J, K, M** and **N**; 0.1 mm for **D** and **I)**.

Head: Head finely and densely punctate. Occiput flat and polished. Temple broad and convex, finely punctate. Mandible very short, upper tooth longer than lower tooth (Fig. 1D). Ocelli slightly large; diameter of median ocellus 1.1 times as long as distance between ocellus and eye; lateral ocellus separated from eye by 0.9 times lateral ocellus diameter.

Mesosoma: Pronotum covered with transverse striae (Fig. 1E); epomia distinct. Mesoscutum finely and densely punctate; notaulus absent (Fig. 1G). Mesopleuron roughly punctate, reticulate; postpectal carina complete; epicnemial carina short; sternaulus absent; speculum convex, impunctate, polished (Fig. 1F); scutellum finely granulate. Propodeum with numerous transverse rugae and reticulate; basal area and areola separated by anterior transverse carina (Fig. 1H); areola and petiolar area not separated, impressed medially; costula complete (Fig. 5A); spiracle very small, round, connected to pleural carina; distance between spiracle and pleural carina 1.7 times as diameter of spiracle (Fig. 1I). Hind tibia with short spines; ratios between hind tarsal segments 4.5: 2.2: 1.6: 0.8: 0.9. Fore wing with areolet; basal vein opposite nervulus (Fig. 1L); nervellus not intercepted; discoidella absent.

Metasoma: Thyridium small, oval; separated from base of 2^nd^ tergite by more than 3.0 times its diameter; distance between base of 2^nd^ tergite and thyridium 0.6 times distance between base of 2^nd^ tergite and spiracle (Fig. 1K). 6^th^ and 7^th^ tergites deeply emarginate apically (Fig. 1M). Ovipositor (Fig. 1N) shorter than metasoma, 1.4 times as long as hind tibia.

Male. Unknown.

##### Distribution.

Korea (new record), Japan, Russia (Khabarovsk, Primor’ye Kray).

#### Cymodusa (Cymodusa) koreana

Choi & Lee sp. n.

urn:lsid:zoobank.org:act:AEEED4C3-D4B6-4E22-9E00-C20FB416D51E

http://species-id.net/wiki/Cymodusa_koreana

Figs 2 , 5I , 6F

##### Material examined.

Holotype: [Korea] (TD: YNU): 1 female, Unmunsa, Chungdo-gun, GB, Korea, 21.V.1989, I.S. Ye.

Paratypes: [Korea] (TD: YNU): 1 female, Gyeonbongsa, Ganseong, GW, Korea, 22 May 1992, J.W. Lee; 1 female, Mureung Valley, Samhwa-dong, Donghae-si, GW, Korea, 20 September–2 October 2006, MT, J.W. Lee; 1 female, ditto, 28 August-10 September 2006, MT, J.W. Lee; 1 female, Yeungnam Univ., Gyeongsan-si, GB, 13 May 1985, E.S. Kim; 1 female, Mirimsan, Bonghwa-gun, 4 May 1997, J.C. Jeong.

##### Description

(female holotype). Body length 7.3 mm.

Fore wing length 4.1 mm.

Antenna with 31–32 flagellomeres.

*Color*. Head, scape and pedicel black. Mandible yellow, apically brown. Labial and maxillary palps pale yellow. Antenna blackish brown. Mesosoma black. Tegula yellow. Fore leg yellow; mid leg yellowish brown; hind coxa black, trochanter dark brown, trochantellus yellow, femur brown, tibia yellow medially, brown basally and apically, tarsus brown. Metasoma black, tergites apically narrowly reddish brown. Thyridium reddish brown.

##### Morphology.

**Figure 2.**
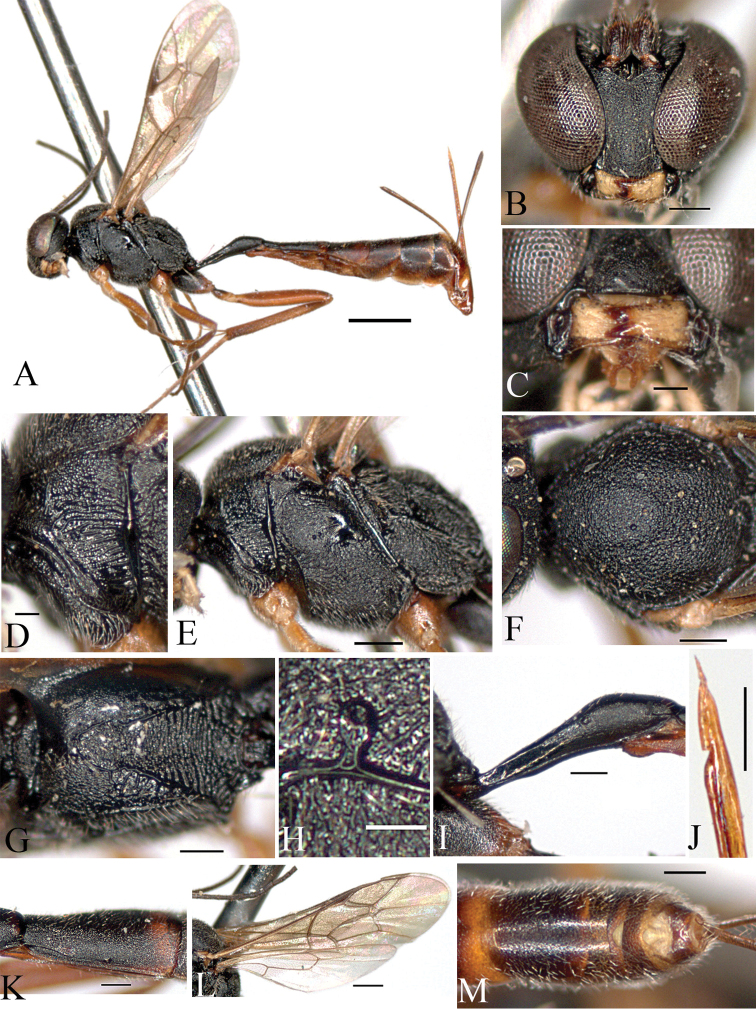
*Cymodusa (Cymodusa) koreana* Choi & Lee sp. n. (female). **A** habitus in lateral view **B** head in frontal view **C** mandible **D** pronotum **E** mesopleuron in lateral view **F** mesoscutum in dorsal view **G** propodeum **H** spiracle of propodeum **I** petiole in lateral view **J** ovipositor **K** thyridium **L** wings **M** 6^th^ and 7^th^ tergites in dorsal view. (Scale bar 1 mm for **A**; 0.5 mm for **L**; 0.2 mm for **B, E–G, I–K** and **M**; 0.1 mm for **C, D** and **H**).

Head: Head finely and densely punctate. Occiput flat and polished. Temple broad and convex, finely punctate. Mandible very short, upper tooth as long as lower tooth (Fig. 2C). Minimum distance between eyes 0.6 times as long as maximum distance (Fig. 2B). Ocelli small; diameter of median ocellus 0.9 times as long as distance between ocellus and eye; lateral ocellus separated from eye by 1.3 times of lateral ocellus diameter. Antenna with 31–32 flagellomeres, 1^st^ flagellomere 1.3 times as long as 2^nd^ flagellomere.

Mesosoma: Pronotum with transverse striae ventrally (Fig. 2D); epomia weak. Mesoscutum finely and densely punctate; notaulus absent (Fig. 2F). Mesopleuron roughly punctate, reticulate; postpectal carinae complete, epicnemial carina short; sternaulus absent. Scutellum finely punctate; postscutellum broader than high. Propodeum with numerous transverse rugae and reticulate; basal area and areola separated by anterior transverse carina; costula absent (Fig. 5I); areola and petiolar area not separated, impressed medially (Fig. 2G); spiracle small, round, connected to pleural carina, distance between spiracle and pleural carina 0.9 times as long as diameter of spiracle (Fig. 2H). Hind tibia with short spines; ratio between hind tarsal segments 5.0: 2.2: 1.5: 0.9: 0.9. Fore wing with areolet; basal vein opposite nervulus; nervellus not intercepted; discoidella absent (Fig. 2L).

Metasoma: Thyridium small, oval; separated from base of 2^nd^ tergite by more than 2.0 times its diameter; distance between base of 2^nd^ tergite and thyridium 0.5 times as long as distance between base of 2^nd^ tergite and spiracle (Fig. 2K). 6^th^ and 7^th^ tergites deeply emarginate apically (Fig. 2M). Ovipositor 1.5 times as long as hind tibia (Fig. 2J).

Male. Unknown.

##### Distribution.

Korea.

##### Etymology.

The specific name is derived from Korea, the locality of the type specimens.

##### Comments.

This species is similar to *Cymodusa rufiventris* Dbar, 1985 in the structure of the propodeum and costula, but the basal area is different, areolet is sessile and pentagonal, and the metasoma is darker.

#### Cymodusa (Cymodusa) yeungnamensis

Choi & Lee sp. n.

urn:lsid:zoobank.org:act:2BF6F784-E72A-411C-BB6D-44DDD147738B

http://species-id.net/wiki/Cymodusa_yeungnamensis

Figs 3 , 5J , 6G

##### Material examined.

Holotype: [Korea] (TD: YNU): 1 female, Yeungnam Univ., Gyeungsan-si, GB, Korea, 21 May 1990, M.J. Kim.

Paratype: [Korea] (TD: YNU): 1 female, Yeungnam Univ., Gyeungsan-si, GB, Korea, 19 June 1992, G.Y. Lee.

##### Description

(female holotype). Body length 6.9 mm.

Fore wing length 4.0 mm.

Antenna with 18+ flagellomeres, apical flagellomeres missing. (antenna with 32 flagellomeres at paratype)

*Color*. Head black. Scape and pedicel blackish brown. Antenna black, except 3 antennal flagellomeres yellow. Mandible yellow, brown apically. Mesosoma black; tegula brown. Fore leg yellowish brown; mid coxa black, brown apically, trochanter and trochantellus yellow, femur to tarsus brown; hind coxa and trochanter black, trochantellus yellow, femur blackish brown, tibia reddish brown, blackish brown basally and apically, tarsus blackish brown. Metasoma blackish brown; petiole black. Thyridium reddish brown. Ovipositor brown.

Morphology.

**Figure 3.**
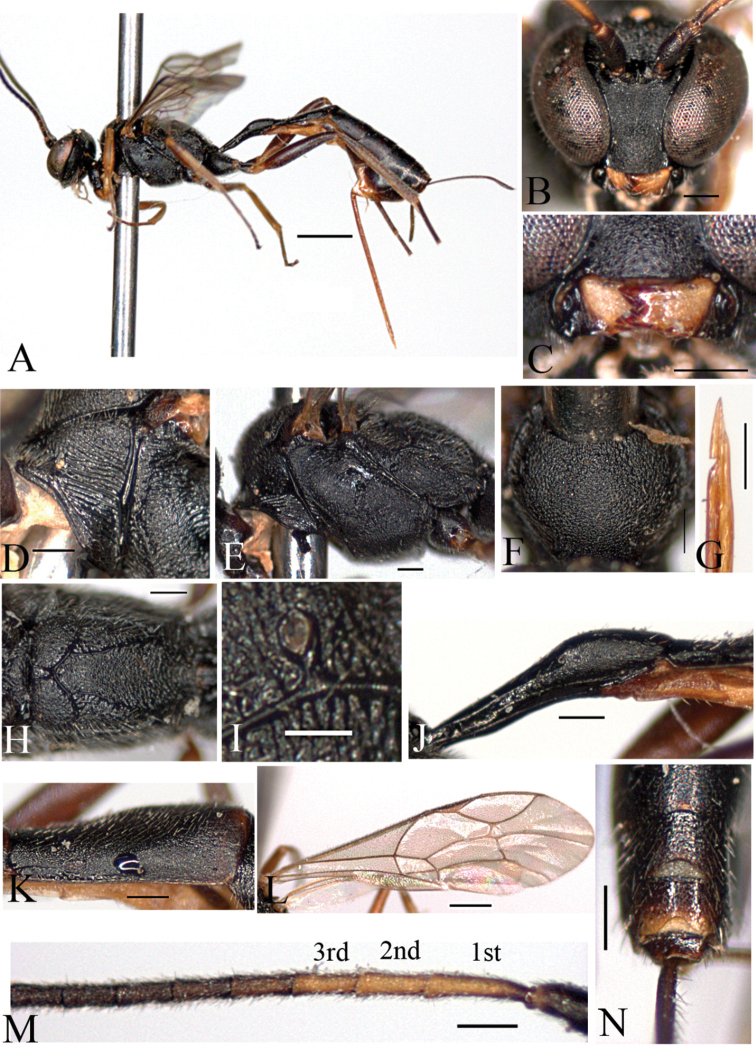
*Cymodusa (Cymodusa) yeungnamensis* Choi & Lee sp. n. (female). **A** habitus in lateral view **B** head in frontal view **C** mandible **D** pronotum **E** mesopleuron in lateral view **F** mesoscutum in dorsal view **G** ovipositor **H** propodeum **I** spiracle of propodeum **J** petiole in lateral view **K** thyridium **L** wings **M** antenna **N** 6^th^ and 7^th^ tergites in dorsal. (Scale bar 1 mm for **A**; 0.5 mm for **L**; 0.2 mm for **B–H, J, K, M** and **N**; 0.1 mm for **I**).

Head: Head densely finely punctate; Vertex slightly punctate. Occiput flat and polished. Temple finely punctate and flat. Mandible very short, upper tooth as long as lower one (Fig. 3C). Minimum distance between eyes 0.6 times as long as maximum distance (Fig. 3B). Ocelli small; diameter of median ocellus 0.8 times as long as distance between ocellus and eye; lateral ocellus separated from eye by 1.6 times of lateral ocellus diameter. Antenna with 32 flagellomeres, 1^st^ flagellomere 1.5 times as long as 2^nd^ flagellomere.

Mesosoma: Pronotum sparsely punctate; upper part reticulate; ventrally with transverse striae (Fig. 3D); epomia absent. Mesoscutum closely and finely punctate; notaulus absent (Fig. 3F). Mesopleuron reticulate; postpectal carinae complete; epicnemial carina reaching anterior margin of mesopleuron at its middle (Fig. 3E); epicnemial carina present; sternaulus absent. Scutellum closely punctate; postscutellum flat, broader than high. Propodeum reticulated; basal area and areola not separated by anterior transverse carina; costula present but incomplete (Fig. 5J); areola and petiolar area not separated, impressed (Fig. 3H); spiracle round; connected to pleural carina; distance between spiracle and pleural carina 0.7 times as long as diameter of spiracle (Fig. 3I). Hind tibia with short spines; ratio between hind tarsal segments 5.1: 2.4: 1.6: 0.9: 1.0. Fore wing with areolet (Fig. 3L); basal vein opposite nervulus; nervellus not intercepted; discoidella absent.

Metasoma: Thyridium separated from base of 2^nd^ tergite by more than 3.0 times its diameter; distance between base of 2^nd^ tergite and thyridium 0.5 times as long as distance between base of 2^nd^ tergite and spiracle (Fig. 3K). 6^th^ and 7^th^ tergites deeply emarginate apically (Fig. 3N). Ovipositor (Fig. 3G) 1.6 times as long as hind tibia.

Male. Unknown.

##### Distribution.

Korea.

##### Etymology.

The specific name is derived from the locality of the type specimens.

##### Comments.

This species is similar to *Cymodusa aenigma* Dbar, 1985, but the areolet is not sessile and the basal 1^st^ to 3^rd^ flagellomeres are yellow below (as in *Cymodusa leucocera* Holmgren and *Cymodusa distincta* Cresson).

#### Cymodusa (Cymodusa) geolimi

Choi & Lee sp. n.

urn:lsid:zoobank.org:act:78152C58-FD90-4622-B380-53174A5C55BC

http://species-id.net/wiki/Cymodusa_geolimi

Figs 4 , 5K , 6H

##### Material examined.

Holotype: [Korea] (TD: YNU): 1 female, Mureung valley, Samhwa-dong, Donghae-si GW, Korea, 16-28 June 2005, MT, J.W. Lee.

Paratype: [Korea] (TD: YNU): 1 female, Gajoa-dong, Jinju-si, GN, Korea, 3-9 June 1989, J.W. Lee.

##### Description

(female holotype). Body length 5.8 mm.

Fore wing length 3.4 mm.

Antenna with 31 flagellomeres.

*Color*. Head black. Scape and pedicel blackish brown. Antenna black. Mandible yellow, brown apically. Mesosoma black; tegula brown. Fore leg yellowish brown; mid coxa black, brown apically, trochanter and trochantellus yellow, femur to tarsus brown; hind coxa and trochanter black, trochantellus yellow, femur blackish brown, tibia reddish brown, blackish brown basally and apically, tarsus blackish brown. Metasoma blackish brown; petiole black. Thyridium reddish brown. Ovipositor brown.

Morphology.

**Figure 4.**
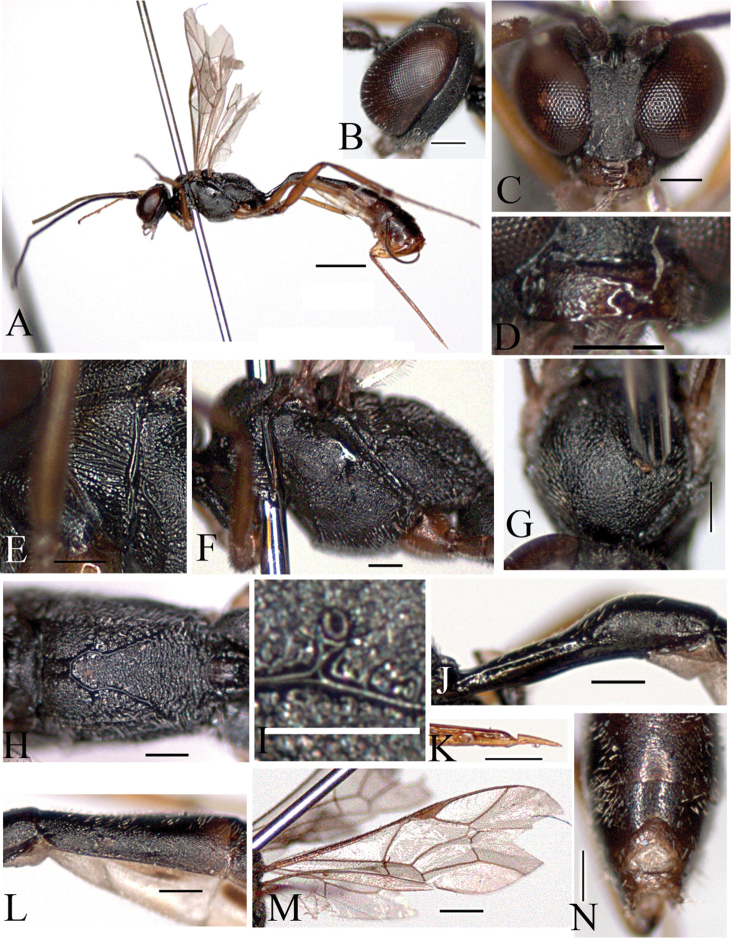
*Cymodusa (Cymodusa) geolimi* Choi & Lee sp. n. (female). **A** habitus in lateral view **B** head in lateral view **C** head in frontal view **D** mandible **E** pronotum **F** mesopleuron in lateral view **G** mesoscutum in dorsal view **H** propodeum **I** spiracle of propodeum **J** petiole in lateral view **K** ovipositor **L** thyridium **M** wings **N** 6^th^ and 7^th^ tergites in dorsal view. (Scale bar 1 mm for **A**; 0.5 mm for **M**; 0.2 mm for **B–L** and **N**).

Head: Head closely and finely punctate. Vertex slightly punctate. Occiput flat and polished. Temple finely punctate and flat. Mandibles short, upper tooth as long as lower one. Minimum distance between eyes 0.6 times as long as maximum distance (Fig. 4C). Ocelli small; diameter of median ocellus 0.9 times as long as distance between ocellus and eye; lateral ocellus separated from eye by 1.0 times lateral ocellus diameter. Antenna with 31 flagellomeres, 1^st^ flagellomere 1.3 times as long as 2^nd^ flagellomere.

**Figure 5.**
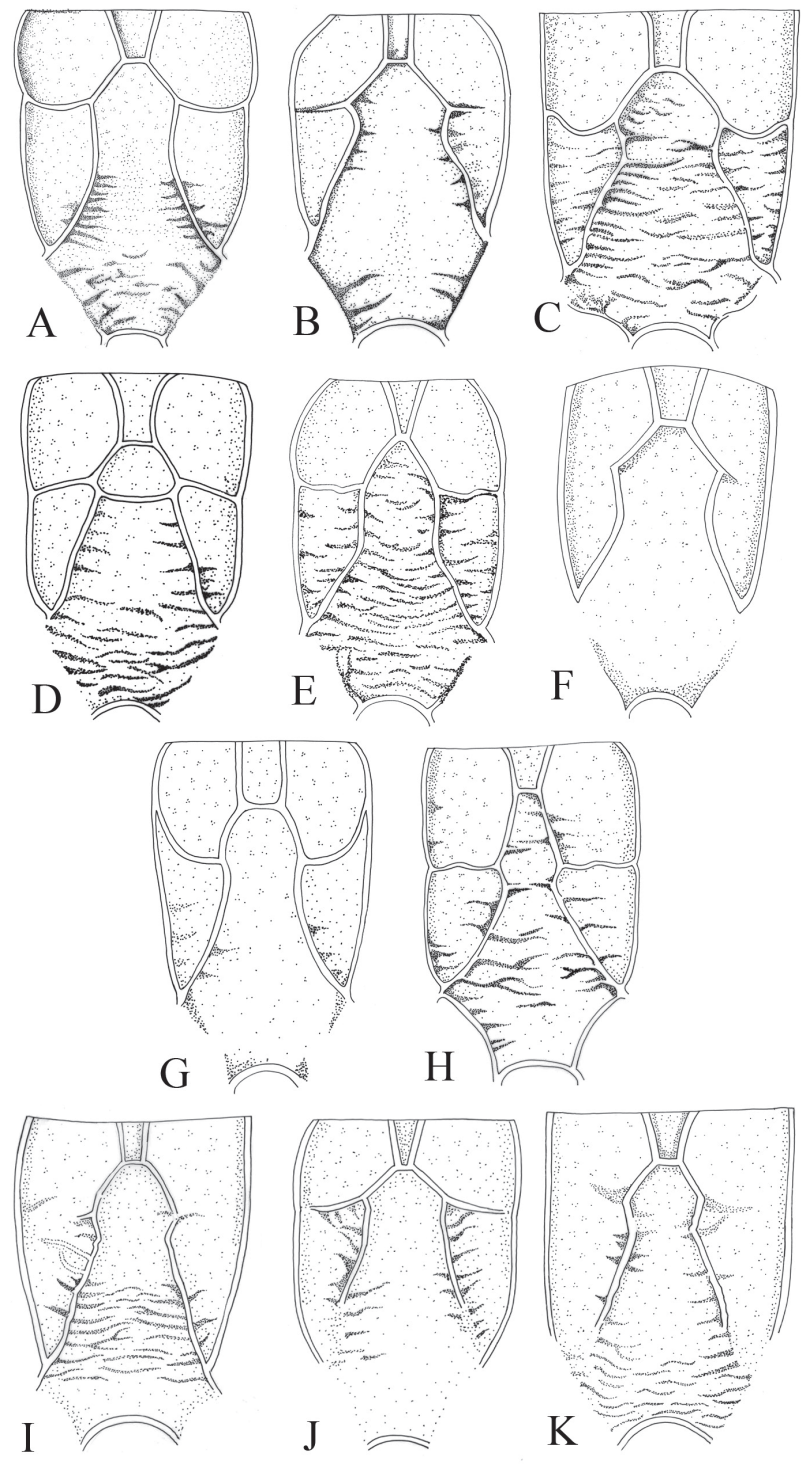
Propodeum, dorsal, of “*australis*” group. **A** *Cymodusa aenigma* **B** *Cymodusa australis* **C** *Cymodusa longiterebra* **D** *Cymodusa oculator* **E** *Cymodusa orientalis* **F** *Cymodusa parva* **G** *Cymodusa rufiventris* **H** *Cymodusa tibialis* **I** *Cymodusa koreana* sp. n. **J** *Cymodusa yeungnamensis* sp. n. **K** *Cymodusa geolimi* sp. n.

Mesosoma: Pronotum sparsely punctate; upper part reticulated; lower part with transverse striae; epomia absent. Mesoscutum closely and finely punctate; notaulus absent (Fig. 4G). Mesopleuron reticulate (Fig. 4F); postpectal carinae complete; epicnemial carina present; sternaulus absent. Scutellum closely punctate; postscutellum flat, broader than high. Propodeum reticulate; basal area and areola separated by anterior transverse carina (Fig. 5K); costula weak; areola and petiolar area not separated, impressed; spiracle small, round; distance between spiracle and pleural carina 1.00 times diameter of spiracle (Fig. 4I). Hind tibia with short spines; ratio between hind tarsal segments 4.4: 2.0: 1.3: 0.7: 0.9. Fore wing with areolet; basal vein opposite nervulus (Fig. 4M); nervellus not intercepted; discoidella absent.

**Figure 6.**
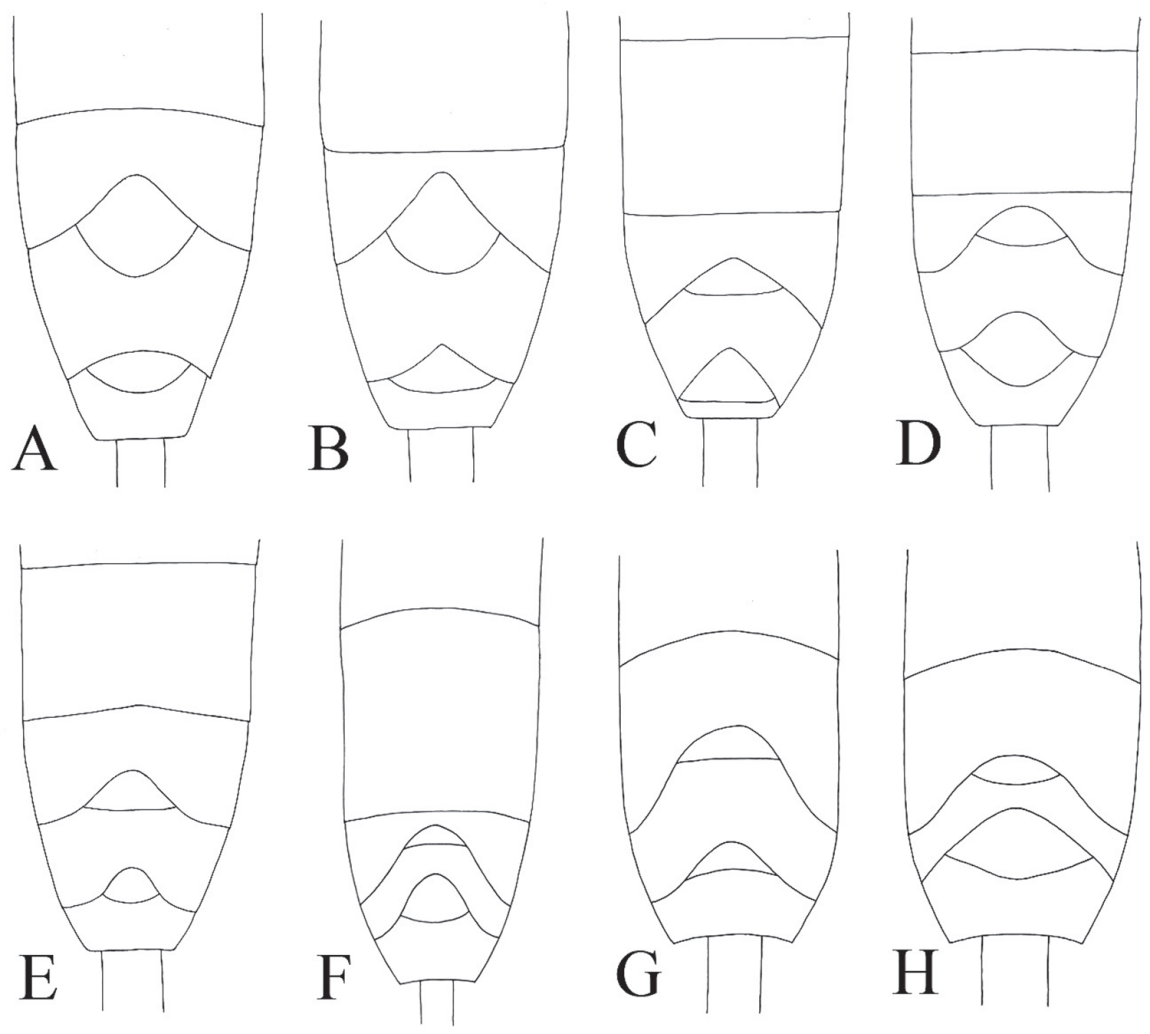
6^th^ and 7^th^ metasomal terga, dorsal, of “*australis*” group. **A** *Cymodusa aenigma* **B** *Cymodusa longiterebra* **C** *Cymodusa orientalis* **D** *Cymodusa rufiventris* **E** *Cymodusa tibialis* **F** *Cymodusa koreana* sp. n. **G** *Cymodusa yeungnamensis* sp. n. **H** *Cymodusa geolimi* sp. n.

Metasoma: Thyridium separated from base of 2^nd^ tergite by more than 4.0 times its diameter; distance between base of 2^nd^ tergite and thyridium 0.6 times as long as distance between base of 2^nd^ tergite and spiracle (Fig. 4L). 6^th^ and 7^th^ terga deeply emarginate apically (Fig. 4N); 7^th^ tergite longer than 6^th^ tergite (Fig. 6H). Ovipositor (Fig. 4K) shorter than metasoma, 1.7 times as long as hind tibia.

Male. Unknown.

##### Distribution.

Korea.

##### Etymology.

The species is named after the nickname of Dr. Jong-Wook Lee, who collected the type specimens.

##### Comments.

This species is similar to *Cymodusa oculator* Dbar (1985) but differs by the number of flagellar segments, developed clypeal fovea, basal area and areola not separated, and a different color pattern.

## Supplementary Material

XML Treatment for Cymodusa (Cymodusa)

XML Treatment for Cymodusa (Cymodusa) aenigma

XML Treatment for Cymodusa (Cymodusa) koreana

XML Treatment for Cymodusa (Cymodusa) yeungnamensis

XML Treatment for Cymodusa (Cymodusa) geolimi
